# Correction: CO_2_ free production of ethylene oxide *via* liquid phase epoxidation of ethylene using niobium oxide incorporated mesoporous silica material as the catalyst

**DOI:** 10.1039/d3ra90009f

**Published:** 2023-02-09

**Authors:** Muhammad Maqbool, Toheed Akhter, Muhammad Faheem, Sohail Nadeem, Chan Ho Park

**Affiliations:** a Department of Chemistry, University of Management and Technology C-II, Johar Town Lahore 54770 Pakistan Toheed.akhter@umt.edu.pk; b Department of Chemical and Biological Engineering, Gachon University Seongnam 13120 Republic of Korea chhopark@gachon.ac.kr

## Abstract

Correction for ‘CO_2_ free production of ethylene oxide *via* liquid phase epoxidation of ethylene using niobium oxide incorporated mesoporous silica material as the catalyst’ by Muhammad Maqbool *et al., RSC Adv.*, 2023, **13**, 1779–1786, https://doi.org/10.1039/D2RA07240H

The authors regret that the inclusion of author Asif Mahmood in the author list and author contributions statement of the original manuscript was incorrect.

Asif Mahmood and their affiliation have been removed from the author list. The corrected author list and list of affiliations is shown here.

The updated author contributions statement is as follows:

Conceptualization, Toheed Akhter; methodology, Muhammad Maqbool, Toheed Akhter; formal analysis, Muhammad Maqbool and Toheed Akhter; investigation, Muhammad Maqbool; resources, Sohail Nadeem, Muhammad Faheem and Chan Ho Park; writing—original draft preparation, Toheed Akhter and Muhammad Maqbool; writing—review and editing, Toheed Akhter and Chan Ho Park; supervision, Toheed Akhter; all authors have read and agreed to the published version of the manuscript.

The Royal Society of Chemistry apologises for these errors and any consequent inconvenience to authors and readers.

## Supplementary Material

